# Genomic comparison of serogroups O159 and O170 with other *Vibrio cholerae* serogroups

**DOI:** 10.1186/s12864-019-5603-7

**Published:** 2019-03-25

**Authors:** Zhenpeng Li, Xin Lu, Duochun Wang, Wei Li Liang, Jingyun Zhang, Jie Li, Jialiang Xu, Bo Pang, Biao Kan

**Affiliations:** 1 State Key Laboratory for Infectious Disease Prevention and Control, National Institute for Communicable Disease Control and Prevention, Chinese Center for Disease Control and Prevention, Beijing, 102206 China; 2 Collaborative Innovation Center for Diagnosis and Treatment of Infectious Diseases, Hangzhou, 310003 China; 3 Jialiang Xu, Key Laboratory of Cleaner Production and Integrated Resource Utilization of China National Light Industry, School of Food and Chemical Engineering, Beijing Technology and Business University, Beijing, 100048 China

**Keywords:** *Vibrio cholerae*, Serogroup, Virulence-related gene, Selection pressure

## Abstract

**Background:**

Of the hundreds of *Vibrio cholerae* serogroups, O1 and O139 are the main epidemic-causing ones. Although non-O1/non-O139 serogroups rarely cause epidemics, the possibility exists for strains within them to have pathogenic potential.

**Results:**

We selected 25 representative strains within 16 *V. cholerae* serogroups and examined their genomic and functional characteristics. We tentatively constructed a gene pool containing 405 homologous gene clusters, which is well organized and functions in O-antigen polysaccharide (O-PS) synthesis. Our network analysis indicate that great diversity exists in O-PS among the serogroups, and several serogroup pairs share a high number of homologous genes (e.g., O115 and O37; O170 and O139; O12 and O39). The phylogenetic analysis results suggest that a close relationship exists between serogroups O170, O89 and O144, based on neighbor-joining (NJ) and gene trees, although serogroup O159 showed an inconsistent phylogenetic relationship between the NJ tree and the gene tree, indicating that it may have undergone extensive recombination and horizontal gene transfer. Different phylogenetic structures were observed between the core genes, pan genes, and O-PS genes. The virulence gene analysis indicated that the virulence genes from all the representative strains may have their sources from four particular bacteria (*Pseudomonas aeruginosa*, *V. vulnificus*, *Haemophilus somnus* and *H. influenzae*), which suggests that *V. cholerae* may have exchanged virulence genes with other bacterial genera or species in certain environments. The mobile genetic element analysis indicated that O159 carries nearly complete VSP-II and partial VPI-1 and VPI-2, O170 carries partial VPI-1 and VPI-2, and several non-O1/non-O139 strains contain full or partial VPI-1 and VPI-2. Several genes showing evidence of positive selection are involved in chemotaxis, Na + resistance, or cell wall synthesis, suggestive of environmental adaptation.

**Conclusions:**

This study reports on the newly sequenced O159 and O170 genomes and their comparisons with other *V. cholerae* serogroups. The complicated O-PS network of constituent genes highlights the detailed recombination mechanisms that have acted on the serogroups’ genomes. The serogroups have different virulence-related gene profiles, and there is evidence of positive selection acting on other genes, possibly during adaptation to different environments and hosts.

**Electronic supplementary material:**

The online version of this article (10.1186/s12864-019-5603-7) contains supplementary material, which is available to authorized users.

## Background

Lipopolysaccharide (LPS) is vital to the structural and functional integrity of the outer membrane of Gram-negative bacteria. LPS is also one of the primary targets of the innate arm of the mammalian immune system [1]. As one of the three LPS constituents, O-antigen polysaccharide (O-PS) in all bacterial serogroups has a distinct structure. Hundreds of serotypes or serogroups can be classified by the diversity of O-PS in individual Gram-negative species.

On the basis of the variable somatic O antigen composition, 206 serogroups of *Vibrio cholerae* have been recognized [2], but not all of them cause the severe diarrheal disease known as cholera. Seven cholera pandemics have been reported throughout history. Based on the pathogenic evidence, the sixth and seventh cholera pandemics were caused by *V. cholerae* serogroup O1, a pathogen with the ability to produce cholera toxin (CT). Genome sequencing has also confirmed that the *V. cholerae* obtained from the preserved intestines of a Philadelphian victim of the second cholera pandemic in 1849 belongs to toxigenic serogroup O1 [3]. *V. cholerae* serogroup O1 has both toxigenic and non-toxigenic strains, among which the genomes of the toxigenic strains are highly clonal, whereas great divergence is found in the non-toxigenic strains [4, 5]. In addition to serogroup O1, epidemics caused by the O139 toxigenic serogroup have also occurred in Southeast Asia after 1992 [6]. Other than serogroup O139, serogroup O37 caused a local cholera outbreak in Sudan in 1968 [7]. To date, serogroup O1 remains the main serogroup to cause cholera outbreaks and epidemics, although the El Tor biotype and its variant strains have been involved in numerous outbreaks around the world and have become prevalent in some countries, such as Haiti, Yemen and South Africa [8–10].

Serogroup non-O1/non-O139 refers to the strains that do not agglutinate with O1- or O139-specific antisera but are morphologically and biochemically indistinguishable from serogroup O1. The O-PS structure of *V. cholerae* from any serogroup is unique [11]. In *V. cholerae* O1, the O-PS genes are organized in a gene cluster between the *gmhD* and *rjg* open reading frames (ORFs), and this cluster is defined as the *wbe* region [12]. The O-PS gene cluster from O1 consists of five regions whose genes encode perosamine biosynthesis, O-antigen transport, tetronate biosynthesis, O-antigen modification, and some additionally required genes [13]. Gene cluster comparisons suggest that O139 probably resulted from a precise 22-kb deletion of the *wbe* region of O1, with replacement by a 35-kb *wbf* region (*wbfA* through *wbfX*) encoding the O139 O-antigen [14], possibly involving a homologous recombination event [15]. Evidence has shown that the emergence of non-O1 /non-O139 *V. cholerae* strains with pathogenic potential was created by exchange of the O-Antigen biosynthesis region [16]. It has also been proposed that a serogroup O22 strain might have been the donor of the O139-specific genes in the gene transfer event that led to the origin of the O139 serogroup from a progenitor El Tor strain of *V. cholerae* O1 [15].

Analysis of the genomes of the different *V. cholerae* serogroups has strong potential to provide a clearer picture of the population structure of this bacterium. Specifically, analysis of the O-PS gene clusters may reveal the genetic basis of the differential antigen synthesis observed in *V. cholerae*, which gives rise to its different serogroups. Although the genetic variability and origins of the O-PS cluster genes from O1 and O139 are well described [13], far less is known about the non-O1/non-O139 strains. Here, we sequenced the draft genomes of three *V. cholerae* strains from serogroups O159 and O170 whose genomes have not been reported previously. The sequence data from these strains were combined with 28 representative publicly available genome sequences from other serogroups, and the genetic compositions of the O-PS gene clusters were compared. We also describe the genetic characteristics of the representative strains from the different serogroups.

## Results

### Genome sequences of the O159 and O170 strains and summary comparisons of them with other serogroups

**Fig. 1:**
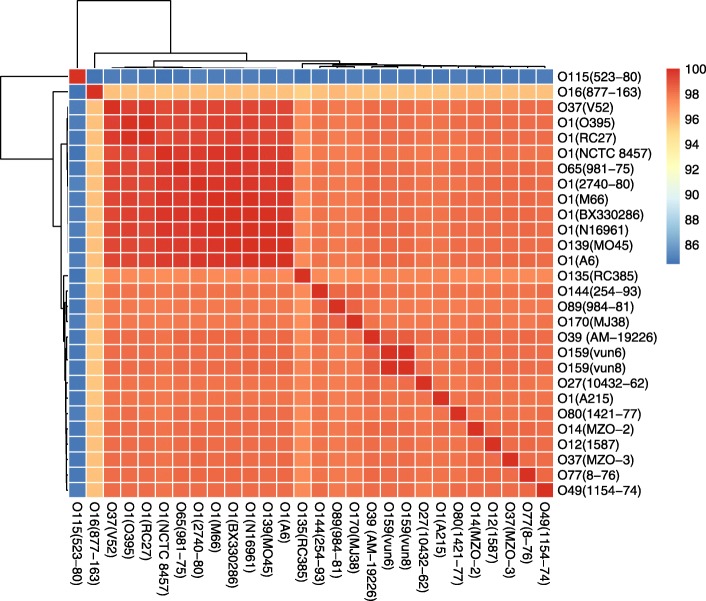
Average nucleotide identity scores for the whole genomic sequences of the selected strains

We sequenced the genomes of two serogroup O159 strains (vun6 and vun8) and one O170 strain (MJ38). We also selected 25 representative strains covering 16 serogroups whose genome sequences and serogroup information could be used for comparing the overall genomic contents and O-PS gene clusters (Table 1). The genome sizes for these strains ranged from 3.78 Mb to 4.25 Mb, and the GC content ranged from 46.13 to 47.77%. The number of ORFs in all the serogroups ranged from 3436 to 3950. Average nucleotide identity (ANI) has been widely used to classify and evaluate the evolutionary distance of prokaryotes at the genomic level [17]. We computed the ANIs for the whole genomic sequences from the serogroups used in this study (Fig. 1). The ANI scores between each of the serogroups ranged from 84.45 to 99.95%. The ANI scores between the two O159 serogroups and the other serogroups ranged from 84.76 to 98.68%, while for the O170 serogroup, they ranged from 84.89 to 98.48%. A large ANI was found between serogroup O39 and O159, while O89 shared the largest ANI with O170.

**Table 1:** Detailed information for the selected strains

Strain	Serogroup	Clade	Country	Year	BioSample ID
O395	O1	Classical	India	1965	SAMN02603898
RC27	O1	Classical	Indonesia	1991	SAMN02393826
A6	O1	L2	Sulawesi	1957	SAMEA889336
N16961	O1	L2	Bangladesh	1975	SAMN02603969
BX330286	O1	L8	Australia	1986	SAMN02393811
M66	O1	L5	Sulawesi	1937	SAMN02603897
NCTC 8457	O1	L6	Saudi Arabia	1910	SAMN02435855
2740–80	O1	L3	USA	1980	SAMN02943003
A215	O1	L4	Australia	1986	SAMEA889299
MO45	O139	–	India	1992	SAMN02693883
vun6	O159	–	China	2010	SAMN08730469
vun8	O159	–	China	2010	SAMN08730470
MJ38	O170	–	China	2007	SAMN08730471
8–76	O77	–	India	1976	SAMN02693891
984–81	O89	–	India	1981	SAMN02693893
1421–77	O80	–	India	1977	SAMN02693892
981–75	O65	–	India	1975	SAMN02693890
10,432–62	O27	–	–	1962	SAMN03248306
877–163	O16	–	Bangladesh	2002	SAMN03580968
MZO-3	O37	–	Bangladesh	2001	SAMN02435869
V52	O37	L7	Sudan	1968	SAMN02435881
1154–74	O49	–	India	1974	SAMN03248305
254–93	O144		India	1993	SAMN02693885
RC385	O135	–	Philippines	1962	SAMN02435834
523–80	O115	–	United States	1980	SAMN02693882
AM-19226	O39	–	Bangladesh	2001	SAMN02435859
1587	O12	–	Peru	1994	SAMN02435867
MZO-2	O14	–	Bangladesh	2001	SAMN02435870

Note: Partial data was collected from NCBI in March 2017

### Organization of the O-PS gene cluster and sharing among the 18 serogroups

**Fig. 2:**
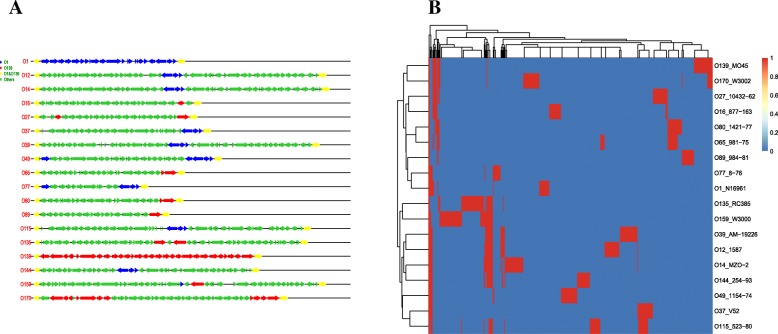
**a**. Structure of the O-PS gene clusters in the different serogroups. Serogroup O1 genes are marked in blue and serogroup O139 genes in red. Their homologous genes in the other serogroups are also marked in the corresponding colors. **b**. Two-dimensional hierarchical clustering of the representative strains with respect to the homologous genes from all the O-PS genetic regions

The genes relating to O-PS biosynthesis were predicted in the genomes of the selected strains. All the genetic regions in the O-PS were flanked by *gmhD* and *rjg*. The O-PS gene clusters for the strains used in this study are listed in Fig. 2a. These clusters showed vast differences among the serogroups. The detailed information for each gene, such as its position in the O-PS genetic regions and the functional annotations of the genes, are listed in Additional file 1. The number of genes within the clusters ranged from 16 to 48. Both serogroup O159 strains had 48 ORFs within their O-PS gene clusters, while the O170 strain had 38. Through use of a homologous relationship analysis with all the O-PS genetic regions from the different serogroups, 405 homologous gene clusters were found in the 18 serogroups (Fig. 2b). These homologous genes may comprise the organized and functional gene pool for O-PS synthesis in *V. cholerae*. As O1 and O139 are the only two serogroups that can cause cholera epidemics, we examined the O-antigen genes in O1 and O139 that are present in the other serogroups. A three-gene sub-cluster in serogroup O1, which contains the genes encoding a polysaccharide biosynthesis protein, an NAD-dependent dehydratase and the lipid carrier UDP-N-acetylgalactosaminyltransferase, were also found to exist in eight serogroups (Fig. 2), suggesting they may have functional relevance. For the O-PS genes from serogroup O139, seven other serogroups had one or two homologous genes, whereas O170 shared 13 homologous genes with O139, covering two sub-clusters (Fig. 2). O159 shared three gene homologs with O139, annotated as ADP-L-glycero-D-manno-heptose-6-epimerase, transposase and conserved hypothetical protein.

**Fig. 3:**
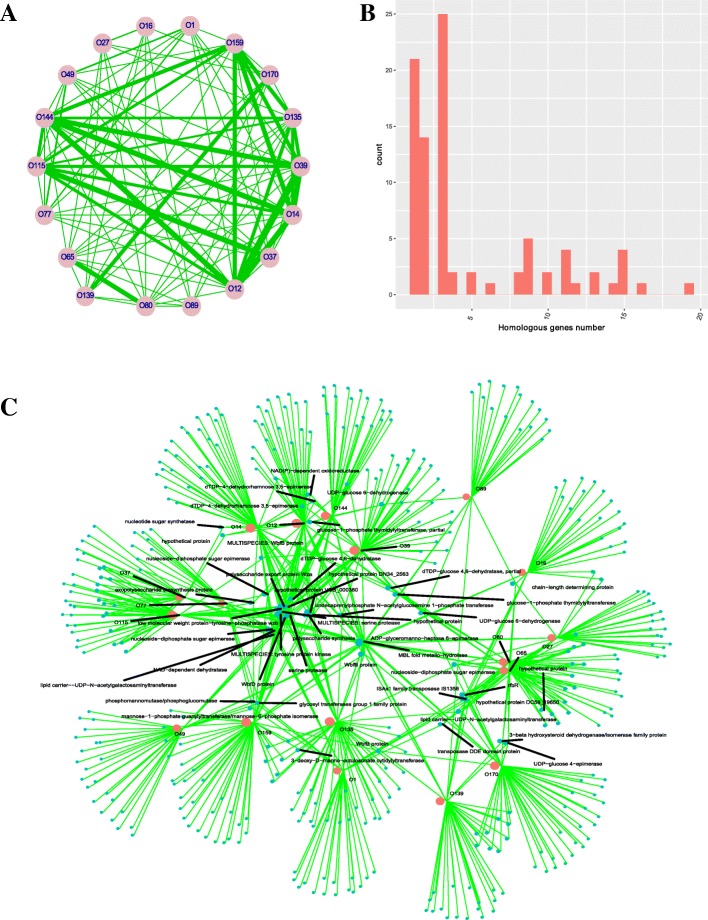
**a**. Relationship network for different serogroups based on homologous gene sharing. **b**. Distribution of the number of serogroups shared by any homologous gene. **c**. Relationship network for the different serogroups and their genes in the O-PS genetic regions

A relationship network for the different serogroups based on homologous gene sharing was constructed. The width of links was proportional to the number of shared O-antigen synthesis genes (except the flanking genes, *rgj* and *gmhD*). Our 2-dimensional hierarchical clustering and network analysis revealed the global O-antigen region similarity levels between the different serogroups (Figs. 2b and 3a). The highest number of homologous genes was shared by O65 and O80. Several serogroup pairs (O115/O37, O170/O139, and O12/ O39) shared large numbers of homologous genes. The distribution of the homologous genes shared by any two serogroups is shown in Fig. 3b. The distribution was found to display a typical power-law distribution. Most serogroup pairs shared relatively few homologous genes, with a few sharing more of these genes. This distribution further confirmed the diverse sources from which the O-PS gene cluster arose. Collectively, these results indicate that there is great diversity in the O-antigen gene cluster among the serogroups.

We also constructed a relationship network for the different serogroups and their O-PS genes. The results of this network analysis revealed the detailed distribution of the homologous gene clusters in the different serogroups (Fig. 3c). Several genes were widely distributed across several serogroups, such as UDP-N-acetylgalactosaminyltransferase, NAD-dependent dehydratase and nucleoside-diphosphate sugar epimerase. The detailed annotation and distribution of the homologous genes in the different serogroups are listed in Additional file 2.

### Coevolutionary analysis of the O-PS gene clusters and the core genomes of the strains

**Fig. 4:**
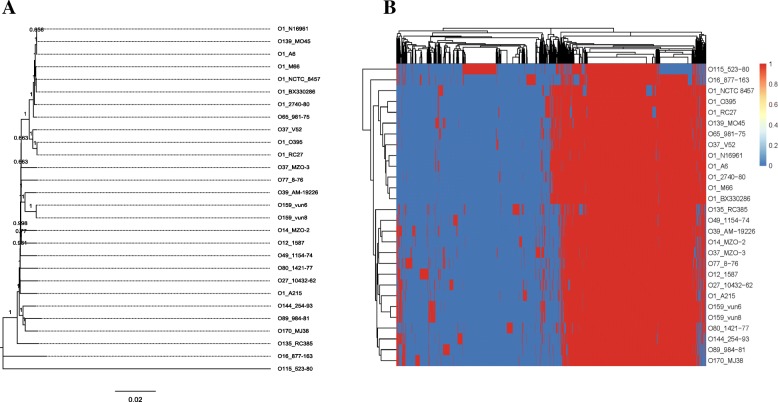
**a**. Neighbor-joining tree for representative *V. cholerae* strains including serogroups O159 and O170. **b**. Gene tree for representative *V. cholerae* strains with respect to the homologous genes from all the genomes

To determine whether the O-PS gene clusters from the serogroups have co-evolved with the core genomes from these groups, a neighbor-joining (NJ) tree was constructed based on all the core genes from the strains used in this study (Fig. 4a). The seventh-pandemic O1 strains and the toxigenic O139 strain clustered together in the NJ tree. The Indian O65 strain (981–75) also shared a genetically close relationship with the epidemic strains. A closer cluster comprising the toxigenic O1 classical biotype strains and the Sudan outbreak serogroup O37 strain was identified. Other non-O1/non-O139 strains and one non-toxigenic O1 strain (A215) differed extensively from the clusters containing the toxigenic strains. Two O159 strains clustered with O39, and O170 clustered with O89 and O144. From the gene tree (Fig. 4b), O170 also clustered with O89 and O144 strains, while O159 clustered with O1 (A215) and O27, thus revealing an inconsistent topology in the NJ tree.

The NJ tree and gene tree for the various serogroups was strikingly different to the O-PS gene tree (Fig. 2b). Therefore, we developed a method to quantitatively describe these differences. First, we sampled one representative strain from each serogroup, and then computed the pair-wise distances for all of the sampled strains. Therefore, for one strain (A), we picked the strain (B) with the nearest genetic distance in terms of the O-antigen region. We then ranked all the strains according to their distances to A for the whole-genome phylogeny and found the rank of B. Finally, we obtained a rank for each serogroup. The mean rank was 17.56 for the comparison between the core genes and the O-PS genetic regions. The mean rank decreased to 14.47 for the comparison between the pan-genes and the O-antigen regions. Compared with the core genes, the pan-genes had slightly increased (*p* = 0.2176, t-test) evolutionary consistency with the O-PS genetic regions. Based on the pair-wise distances, two pairs of serogroups showed consistent relationships between the core gene-based and the O-PS genetic region-based distance, they were O12_1587/O14_MZO-2 and O12_1587/O39_AM-19,226; and two serogroup pairs showed consistent relationships between the pan gene-based and the O-PS genetic region-based distance, they were O39_AM-19,226/O12_1587 and O115_523–80/O37_V52 (data not shown).

### Distribution of the virulence-related genes and typical mobile genetic elements in the different serogroups

The Virulence Factors Database (VFDB) [18] was used to annotate the virulence-related genes from the selected strains in this study. The number of virulence-related genes ranged from 507 to 577 in the studied strains. Compared with the other strains, O159 and O170 had 9 and 17 serogroup-specific virulence genes, respectively. The number of serogroup-specific virulence-related genes ranged from 1 to 100 in all the serogroups. The serogroup-specific virulence genes in O159 encode proteins that are involved in lipooligosaccharide and capsule biosynthesis and transport, while those of O170 are involved in O-PS, the capsule, and LPS.

**Fig. 5:**
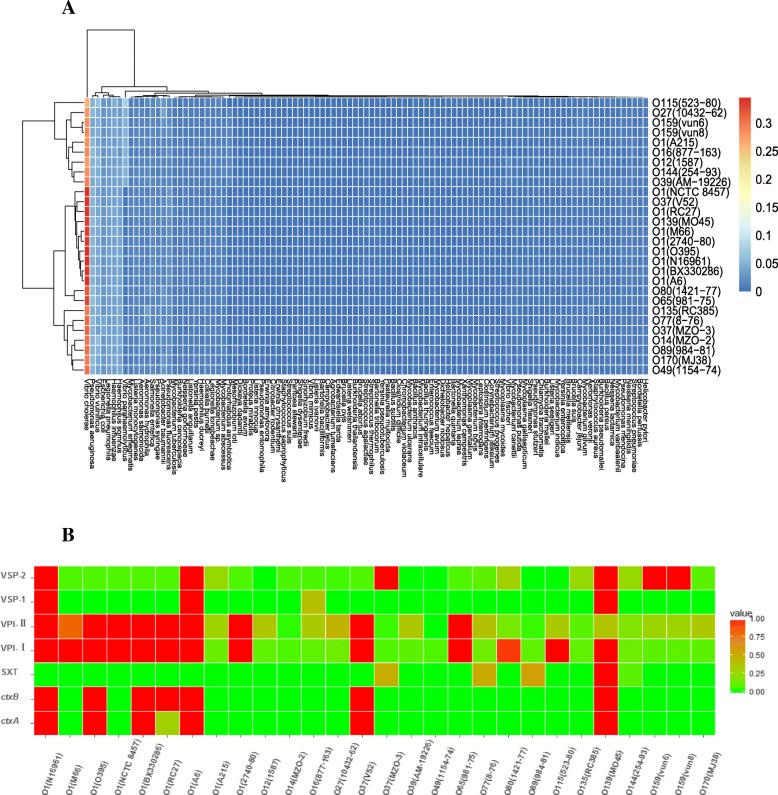
**a**. Two-dimensional hierarchical clustering of the representative *V. cholerae* strains with respect to the virulence gene sources. **b**. Distribution of mobile genetic elements in the different serogroups

Many virulence-related genes in bacteria have been gained by horizontal gene transfer or homologous recombinant during the evolutionary process. Previous research has shown that the virulence regions from four strains of the non-O1/non-O139 serogroups are quite heterogeneous in their compositions [16]. The virulence-related genes in these serogroups may have experienced a wide variety of genetic transfers, making them differ from other species. By searching the virulence-related genes from non-*Vibrio* species, it may be possible to discern whether such genes have close evolutionary relevance to the virulence genes transferred from other species. We chose a relatively relaxed cutoff (identity 30%, coverage 60%) to do this analysis. The results showed that 103 bacterial species share similar virulence-related genes with the *V. cholerae* strains examined in the present study (Fig. 5a). Among these species, *V. cholerae*, *Pseudomonas aeruginosa*, *V. vulnificus*, *Haemophilus somnus* and *H. influenzae* accounted for a high proportion. The virulence-related genes from the O159 strain were sourced from 80 species, and those of O170 were sourced from 82 species. Most of the serogroup-specific virulence genes from O159 were sourced from the following four species: *Campylobacter jejuni*, *Acinetobacter baumannii*, *Streptococcus suis* and *H. influenzae*. Most of the serogroup-specific virulence genes from O170 were sourced from several species, including *A. baumannii*, *C. jejuni*, and *Yersinia pestis* among others. Detailed information on the serogroup-specific virulence genes from the different serogroups can be found in Additional file 3. When a more stringent cutoff (identity 90%, coverage 80%) was used, the virulence-related genes from the 18 serogroup strains shared close relationships with four species: *V. cholerae*, *V. parahaemolyticus*, *V. vulnificus* and *V. mimicus*.

We also analyzed the existing profiles of several classical mobile genetic elements (i.e., VPI-1, VPI-2, VSP-I, VSP-II, SXT and CT) in the selected strains (Fig. 5b). Contig mapping indicated that O159 had a nearly complete VSP-II sequence and partial sequence for VPI-1 and VPI-2. O170 carried partial VPI-1 and VPI-2 sequences. Neither of these two serogroup strains carried the CT.

### Selection pressure analysis of protein-coding genes

*V. cholerae* is an environmental bacterium that lives in different hosts and habitats. Hence, identifying genes in *V. cholerae* that carry the signature of positive selection and may therefore contribute to adaptation to its changing niche should provide useful information about this pathogen. Here, selection pressure on the *non-O1 V. cholerae* strains was analyzed using *V. cholerae* strain N16961 as the reference strain. Four genes showing evidence of positive selection were obtained for each of the three strains (the two serogroup O159 strains and one O170 strain sequenced in this study). The positively selected genes in the two O159 serogroups encode one methyl-accepting chemotaxis protein and three hypothetical proteins. The positively selected genes in the O170 strain encode one nucleoside triphosphate diphosphatase (mazG) protein and three hypothetical proteins.

**Fig. 6:**
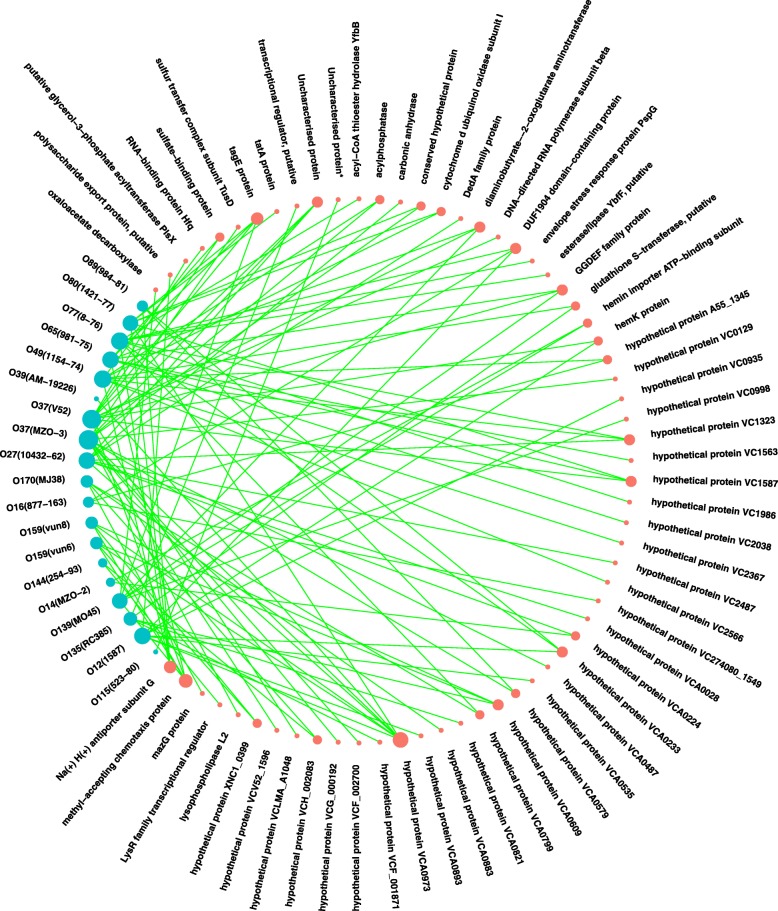
Relationships among the different serogroups and genes showing evidence of positive selection. The nodes representing the serogroups are shown in blue, and the nodes representing the homologous genes are shown in red. The size of each node is proportional to its degree. If the genes appear to have evolved under positive selection in a serogroup, they are linked with that serogroup

To obtain a global view of the positively selected genes from the different serogroups, we constructed a network for the different serogroups and their corresponding positively selected genes, which shows the positively selected genes for each representative strain (Fig. 6). Two O37 strains had the largest number of positively selected genes. O49, O77, O12, O27, O65, O139, O80 and O135 each had more than four positively selected genes, while O16, O89, O14, O144, O115 and O39 each had fewer than four. Taking a general view of the 63 positively selected genes, most of them were found to encode hypothetical proteins. Several genes showed evidence of positive selection in several representative strains. In addition to hypothetical protein genes, the genes encoding the methyl-accepting chemotaxis protein in five strains, Na(+) H(+) antiporter subunit G, and tagE protein in four strains, and diaminobutyrate--2-oxoglutarate aminotransferase, DUF1904 domain-containing protein and GGDEF family protein in three strains were identified as showing evidence of positive selection. Other genes showed evidence of positive selection in fewer than three strains.

## Discussion

Most genomic studies on *V. cholerae* have focused on serogroup O1, but the global genomic landscape for the non-O1/non-O139 serogroups, the genetic diversity of the O-PS gene clusters, and the evolutionary associations with O1 and O139 serogroups have not been adequately studied. Here, we sequenced three strains from two O159 and O170 serogroups, together with 25 strains from 16 other serogroups to reveal their O-PS gene cluster organizations, genomic features and the evolutionary associations among the different serogroups.

The ANI scores between the different strains reflect the level of global similarity among them. Based on our results, serogroups O159 and O39 are most similar in terms of their ANI score, as are serogroups O170 and O89. Among all the representative strains, most share high similarity scores with each other and only serogroup O115 has an ANI score lower than 95% when compared with all the other strains.

O-PS contains genetic signatures that allow the genetic clones of strains within a species to be distinguished. The genes related to O-PS biosynthesis are generally organized together to form a cluster. All the O-PS biosynthesis genes from the 18 serogroups from this study clustered together and were chromosomally located between the *gmhD* and *rjg* genes, suggesting that site-specific transfer has occurred among the different serogroups and strains. Within the O-PS gene clusters, several genes are grouped together and appear in the O-PS gene clusters in the different serogroups, possibly showing that frequent recombination has occurred in these gene clusters. Thus, genetic variability in the O-PS biosynthesis genes, coupled with frequent genetic transfers and recombination may have generated the many serogroups of *V. cholerae*. Serogroups O1 and O139 cause cholera epidemics, but only one gene in the O-PS genetic region of serogroup O159 was found to share sequence homology with a serogroup O1 gene. It is interesting that serogroup O170 shares the most sequence homology in the O-PS genes with O139, suggesting that O139 and O170 O-PS genetic regions have similar sub-structures.

By visualizing their vertices and edges, networks are a useful way of displaying complicated relationships among vertices in an intuitive way, and analysis of a specified network may provide new insights into biological systems. Here, we constructed a relationship network containing different *V. cholerae* serogroups and their associated O-PS genes. Previous preliminary research has shown there is variety in the O-PS genetic region in the different serogroups [19], and our network analysis on the homologous genes in the present study supports the idea that there is great diversity in the O-antigen among different serogroups, a viewpoint supported by our 2-dimensional hierarchical clustering analysis. This analysis made further efforts to decipher the detailed relationship between the different serogroups and the contributions of the constituent O-PS genes in each serogroup. The O-PS gene cluster size and gene count showed great divergence among the serogroups. Except for the left and right junction genes in the wb* regions encoding O-PS synthesis, no genes were shared by all the serogroups, further indicating the genetic diversity of the O-PS in *V. cholerae*. Based on the network analysis, some genes shared by more than two serogroups were identified, showing that exchange and recombination has occurred in the genes and even in the gene clusters among the O-PS genetic regions from the different serogroups. This is further supported by the finding that the O-PS genetic regions comprise the combinations of several smaller gene sets with different origins [20]. Our data have revealed that the O-PS biosynthesis gene pool contains 405 homologous gene clusters, and the network analysis results suggest the hot-spots for serogroups or homologous genes that link other serogroups or homologous genes to a high degree. We suggest that the genes in the gene pool facilitate O-antigen shifts in the different *V. cholerae* serogroups. Serogroup conversion of the O1 recipient by the O139 donor has been validated in the laboratory, and it is proposed that the exchange of serogroup-specific gene clusters could potentially occur between the different O serogroups of *V*. cholerae [21].

Our phylogenetic analysis revealed that most toxigenic O1 *V. cholerae* strains and two classical strains form two separate clusters in the tree. One O139 strain clustered together with most toxigenic O1 *V. cholerae* strains. Two non-O1, non-O139 strains, V52 (O37) and 981–75 (O65), appear in the same cluster with toxigenic O1 *V. cholerae* strains, indicating the possibility of O serogroup conversions from O1 to non-O1/non-O139 [20, 22]. Most of the non-O1/non-O139 strains were distributed in the other part of the tree, a finding consistent with that of a previous study [20]. Furthermore, we investigated whether the O-PS biosynthesis gene clusters had undergone co-evolution with the whole genomes in the different *V. cholerae* serogroups. The genomes and O-PS genes had different phylogenic structures in these strains, revealing that asynchronous genetic variation had occurred during their evolution. We also found that several serogroup pairs had consistent relationships between core-genes or pan-genes and the O-PS genetic region. The consistency between the core gene-based and O-antigen region-based genetic distance indicates that these serogroups had the same common ancestors before gaining similar O-PS genetic regions. In addition, the consistency between the pan gene-based and O-PS genetic region-based distance indicates that these serogroups have undergone some horizontal gene transfer events and acquired similar foreign genes before gaining similar O-PS genetic regions.

The virulence-related genes in the *V. cholerae* strains displayed the highest levels of sequence similarity with those from *P. aeruginosa*, *V. vulnificus*, *H. somnus* and *H. influenzae*. As *V. cholerae* has an aboriginal habitat in the environment, these data suggest that *V. cholerae* can exchange virulence-related genes with the above-named species under certain environmental conditions.

Changes that occur in mobile genetic elements may relate to the emergence, drug resistance, fitness and virulence of a strain. Here, we analyzed the profiles of the mobile genetic elements (i.e., VPI-1, VPI-2, VSP- I, VSP-II, SXT and CT) in the different representative strains. We found O159 had a nearly complete VSP-II and partial VPI-1 and VPI-2 sequences, whereas the O170 strain carried partial VPI- 1 and VPI- 2 sequences. VSP-II is considered to be a genetic marker of epidemic *V. cholerae*; therefore, gaining VSP- II may allow a strain to obtain a survival advantage during epidemics [23]*.* VPI is one of two essential virulence gene clusters in epidemic-causing toxigenic *V. cholerae*. Several non-O1/non-O139 strains contain full or partial VPI-1 and VPI-2 islands, indicating their potential to evolve into epidemic strains*.* Because alteration of the O-antigen in pathogenic strains will boost the emergence of new epidemic strains, a combined description of the O-PS genetic region in the non-O1/non-O139 serogroups and their virulence elements, such as CT and TCP, will provide further information on the genesis mechanism of new emerging strains.

Positive selection is known to be an important aspect of the evolution of many pathogens [24, 25]. Indeed, one study showed that the diversification of clinical and environmental *V. cholerae* isolates from Haiti was driven by positive selection [26]. Selection pressure analysis of strains within the different *V. cholerae* serogroups should provide detailed information on their evolutionary trends. Here, we found that several of the strains share genes that appear to have evolved under positive selection and are involved in environmental adaptation (e.g., chemotaxis, Na^+^ resistance and cell wall synthesis). These functions may represent the common mechanisms used by strains within the different serogroups to adapt to environmental changes. Serogroup-specific selected genes were also found, and some positively-selected genes may exist in more than one serogroup. Thus, the genetic complexity of the different serogroups and strains may reflect their adaptability to different niches.

## Conclusions

We sequenced the genomes of three strains within two *V. cholerae* serogroups and conducted a structure analysis of their O-PS gene clusters and those of 25 strains from 16 other serogroups. We detected an inclusive gene pool responsible for O-PS biosynthesis. There was evidence of frequent recombination between the different serogroups and evidence that independent evolution had occurred in the O-PS gene clusters, compared with the genomic evolution of the serogroups used in this study. The serogroups also had different virulence-related gene profiles and different genes displaying the signatures of evolutionary selection, which may reflect their survival under different environmental and host-related pressures. This study was conducted with 16 *V. cholerae* serogroups, and expanding this number should augment our knowledge of the O-PS gene pool in *V. cholerae*, the structural differences between serogroups, and their roles in the taxonomy and evolution of this bacterium.

## Methods

### Strains and genome sequencing

Strains vun6 and vun8, which were identified as serogroup O159 by the National Institute of Infectious Diseases (NIID), Japan, were isolated from the Minjiang River (Fujian province, China). Strain MJ38, which was isolated from the Pearl River (Guangdong province, China), was identified as serogroup O170 by NIID. All three strains were obtained via the national monitoring of *Vibrio* in China and their genomes have not been reported previously in the scientific literature. The genomic DNA extracted from the three strains using the Wizard@ Genomic DNA Extraction Kit (Madison, WI, Promega, USA) following the manufacturer’s instructions was subjected to 250-bp paired-end whole genome sequencing with 150× coverage using the HiSeq sequencer (Illumina HiSeq2000, San Diego, CA, USA). The sequence data have been deposited in the Sequence Read Archive (SRA) (https://www.ncbi.nlm.nih.gov/sra, BioProject ID: PRJNA438839). SOAPdenovo (Version 2.04) was used to assemble the three strains [27].

We also selected 25 other *V. cholerae* strains for which genome sequences and serogroup information was available (covering 16 serogroups, Table 1). Their genome sequences were retrieved from GenBank (https://www.ncbi.nlm.nih.gov/genbank/).

### ANI analysis

A Perl script based on an algorithm [17] was used to compute the ANIs for the draft sequences from the strains. The ANI was evaluated between the query genome and the reference genome and the mean identity of all BLASTN matches was computed as the ANI value. The computation required that the BLASTN matches should have more than 30% sequence identity and an overall alignable region accounting for at least 70% of their length.

### O-PS genetic region extraction and gene prediction

The genome region flanked by the *gmhD* and *rjg* genes was extracted from the draft genomes as the O-PS genetic region. Prodigal (version 2.60) was used to identify the potential genes in the O-PS genetic regions from all the representative strains [28].

### Network analysis of the O-PS genetic regions

A relationship network for the different serogroups was constructed based on the sharing of homologous genes among the different O-PS genetic regions. We established a procedure to display the network using BLAST+ [29] and the igraph package (version1.1.2) [30] in combination. We first calculated the number of homologous genes shared by any two serogroups. The homologous genes were determined by reciprocal BLAST analysis between the two O-PS generic regions under comparison. The BLAST + E-value and query coverage were set at 0.1e-5 and 80% respectively. Next, the igraph package was used to display the sharing network in the different serogroups. The width of the links was proportional to the number of shared O-antigen synthesis genes.

A relationship network for the different serogroups and their O-PS genes was also constructed by combining several tools. First, the coding sequences from all the different O-PS genetic regions were collected together, and the non-redundant homologous gene set from this collection was analyzed using CD-HIT (version 4.6.6, 2016) [31]. Second, a network was constructed using the non-redundant homologous genes set and the different serogroups as the vertices. The homologous genes were linked to the serogroups if they were sourced from those serogroups. The igraph package was also used to display the network.

### Phylogenetic analysis

The coding sequences from the different serogroups were collected together, and a non-redundant homologous gene set was computed for them using CD-HIT. Next, we searched the homologous genes in the non-redundant homologous gene set for the coding sequences of each strain using BLAST+; here, if the homologous gene for a gene in the non-redundant homologous gene set existed in all the selected strains and had just one copy in each strain, the gene was deemed to be a core gene. The core genes were then aligned and merged, and MEGA (version 7.0.18) was used to construct a NJ tree (bootstraps, 1000) [32].

### Gene tree construction

We designed a procedure using an R script. First, we computed a non-redundant homologous gene set for all the coding sequences from the different serogroups using the same method as described for the phylogenetic analysis. We then constructed a matrix whose rows were the selected strains and whose columns were the non-redundant homologous gene set. When a strain had a homologous gene, 1 was entered in the corresponding position in the matrix. Otherwise, 0 was entered. Last, the matrix was used as the input information with which to construct a heatmap, and the pheatmap package in R was used to draw the heatmap [33].

### Selection pressure analysis

The KaKs_Calculator2.0 was used to compute the selection pressure of orthologous genes from the different representative strains [34]. First, we obtained the core genes from all the selected strains by the same methods as those used for the phylogenetic analysis. Second, paraAT was used to codon-align the core genes between the representative strain and the N16961 reference strain [35]. The results of step 2 were used as the input data for the KaKs_Calculator to compute the nonsynonymous (Ka) and synonymous (Ks) substitution rates (Ka/Ks).

### Virulence gene analysis

The VFDB was used to annotate the virulence genes [18]. All the protein sequences from the representative strains were searched against the VFDB using BLAST+. The potential sources of the virulence genes were annotated by using a relatively relaxed cutoff (identity 30%, coverage 60%) and a more stringent cutoff (identity 90%, coverage 80%). To construct the heatmap for the representative strains and the sources of the virulence genes, we constructed a matrix whose rows were strains and whose columns were the species from which the virulence genes arose. Each value in the matrix indicated the proportion of virulence genes for a species, accounting for all the virulence genes of the strain.

## Additional files

Additional file 1: O-PS genes annotations. The detailed information for each gene in the O-PS genetic regions from the different serogroups, such as its position in the O-PS genetic regions and the functional annotations of the genes. (XLSX 91 kb)

Additional file 2: Characterization of the homologous genes in the different serogroups. The detailed annotation and distribution of the homologous genes in the different serogroups. (XLSX 24 kb)

Additional file 3: Characterization of the serogroup-specific virulence genes. The detailed information on the serogroup-specific virulence genes from the different serogroups. (XLSX 91 kb) (XLSX 23 kb)

## Abbreviations

ANI: Average nucleotide identity
CT: Cholera toxin
Ka/Ks: Nonsynonymous (Ka) and synonymous (Ks) substitution rates
LPS: Lipopolysaccharide
NIID: National Institute of Infectious Diseases
NJ: Neighbor-joining
O-PS: O-antigen polysaccharide
ORFs: Open reading frames
